# Mental Health as a Mediator of the Association Between Educational Inequality and Cardiovascular Disease: A Mendelian Randomization Study

**DOI:** 10.1161/JAHA.120.019340

**Published:** 2021-09-02

**Authors:** Daniel P. Jones, Robyn E. Wootton, Dipender Gill, Alice R. Carter, David Gunnell, Marcus R. Munafò, Hannah M. Sallis

**Affiliations:** ^1^ Department of Population Health Sciences Bristol Medical School University of Bristol UK; ^2^ Division of Population Medicine School of Medicine Cardiff University UK; ^3^ MRC Integrative Epidemiology Unit University of Bristol Oakfield House Bristol UK; ^4^ Department of Epidemiology and Biostatistics Imperial College London London UK; ^5^ Clinical Pharmacology and Therapeutics Section Institute of Medical and Biomedical Education and Institute for Infection and Immunity St George’s University of London UK; ^6^ NIHR Biomedical Research Centre at the University Hospitals Bristol and Weston NHS Foundation Trust University of Bristol UK; ^7^ School of Psychological Science University of Bristol UK; ^8^ Centre for Academic Mental Health Population Health Sciences Bristol Medical School University of Bristol UK

**Keywords:** anxiety, cardiovascular disease, depression, education, Mendelian randomization, Cardiovascular Disease, Epidemiology, Mental Health

## Abstract

**Background:**

Education is inversely associated with cardiovascular disease (CVD). Several mediators of this have been established; however, a proportion of the protective effect remains unaccounted for. Mental health is a proposed mediator, but current evidence is mixed and subject to bias from confounding factors and reverse causation. Mendelian randomization is an instrumental variable technique that uses genetic proxies for exposures and mediators to reduce such bias.

**Methods and Results:**

We performed logistic regression and 2‐step Mendelian randomization analyses using UK Biobank data and genetic summary statistics to investigate whether educational attainment affects risk of mental health disorders. We then performed mediation analyses to explore whether mental health disorders mediate the association between educational attainment and cardiovascular risk. Higher levels of educational attainment were associated with reduced depression, anxiety, and CVD in observational analyses (odds ratio [OR], 0.79 [95% CI, 0.77–0.81], 0.76 [95% CI, 0.73–0.79], and 0.75 [95% CI, 0.74–0.76], respectively), and Mendelian randomization analyses provided evidence of causality (OR, 0.72 [95% CI, 0.67–0.77], 0.50 [95% CI, 0.42–0.59], and 0.62 [95% CI, 0.58–0.66], respectively). Both anxiety and depression were associated with CVD in observational analyses (OR, 1.63 [95% CI, 1.49–1.79] and 1.70 [95% CI, 1.59–1.82], respectively) but only depression showed evidence of causality in the Mendelian randomization analyses (OR, 1.09; 95% CI, 1.03–1.15). An estimated 2% of the total protective effect of education on CVD was mediated by depression.

**Conclusions:**

Higher levels of educational attainment protect against mental health disorders, and reduced depression accounts for a small proportion of the total protective effect of education on CVD.

**Nonstandard Abbreviations and Acronyms**
CARDIoGRAMplusC4D: Coronary Artery Disease Genome‐Wide Replication and Meta‐Analysis (CARDIoGRAM) Plus the Coronary Artery Disease (C4D) Genetics
IVW: inverse‐variance weighted
MR: Mendelian randomization

**Clinical Perspective**

**What Is New?**

- We report a negative association between educational attainment and mental health conditions, with Mendelian randomization methods providing evidence for a causal effect.
- We report a positive association between depression and cardiovascular disease with Mendelian randomization methods providing evidence for a causal effect.
- Using conventional observational epidemiology and Mendelian randomization, we estimate that 2% of the protective effect of education on cardiovascular disease is mediated by depression; a sizeable proportion of this mediating effect by depression could be caused by tobacco smoking.

**What Are the Clinical Implications?**

- This study provides further evidence for the complex interplay of physical health, mental health, and social determinants regarding cardiovascular disease.
- This study also provides further evidence for the importance of addressing smoking behavior in the context of clinical depression.
- Investment in educational attainment is likely to be beneficial for both mental health and cardiovascular health; clinical interventions that target mental health could be beneficial for cardiovascular health, especially for individuals in lower socioeconomic positions.

Cardiovascular disease (CVD) is a leading cause of global morbidity and mortality.^1^ The association between socioeconomic inequality and CVD is well‐established, with individuals living in deprived areas typically having much higher cardiovascular mortality than those in less deprived areas.^2^ More specifically, disparities in educational attainment have recently been shown to affect CVD using both conventional and genetic epidemiological techniques.^3^ CVD risk was estimated to decrease by a third for every 3.6 years of additional full‐time education past the age of 11 years.^4^

Several well‐recognized cardiovascular risk factors appear to act as mediators for education.^5^ For example, low educational attainment is associated with increased tobacco smoking, higher body mass index, and higher blood pressure, which, in turn, are linked to increased risk of CVD.^6^ However, after accounting for these mediators, there is a significant proportion of the association between education and CVD that remains unaccounted for.

Existing evidence suggests an association between mental health and CVD as well as an association between education and mental health.^7^, ^8^ Consequently, mental health may be another potential mediator between education and CVD. For example, educational inequality may lead to disparities in psychological development by affecting exposure to stressors, resources to cope with stress, and negative external societal perception.^9^ Poor mental health could, in turn, lead to increased CVD risk with proposed mechanisms including chronic inflammation,^10^ increased sympathetic nervous system activation,^11^ and increased cortisol exposure.^12^

Conventional observational studies that have explored education, mental health, and CVD have, so far, proved inconclusive.^13^, ^14^ Furthermore, observational evidence can be subject to bias from confounding and reverse causation, and, specifically in the context of mediation studies, to measurement error and collider bias.^15^

Mendelian randomization (MR) is an instrumental variable technique that uses genetic variants as a proxy for an exposure of interest.^16^ These variants are randomized at conception and therefore largely inherited independently from other variants affecting confounding factors. These variants are also unchanged throughout the lifetime and so are unlikely to be affected by outcomes of interest, therefore reducing bias from reverse causation. Thus, MR reduces the sources of bias that limit causal inference in conventional observational research and is an effective, complementary tool in investigating potential causality.

The core assumptions of MR are that the genetic variants must be associated with the exposure under investigation, must only affect the outcome via that exposure, and must not share any common causes with the outcome. Provided that these assumptions are met, then an average causal effect can be estimated for genetic compliers in the sample, ie, patients whose exposure status reliably depends on particular genetic variants. The direction of these effect estimates can then be used as evidence against a causal null hypothesis.

Here, we specifically used 2‐step MR, employing summary data for each step. Two‐step MR estimates the exposure‐mediator, exposure‐outcome, and mediator‐outcome effects separately and is particularly useful when mediators are binary. We also utilized separate samples for the instrument and outcome summary data, thus providing greater sample sizes in some cases.

In this study, we used a complementary approach utilizing both observational and MR techniques to explore the relationship between education, mental health, and CVD. Elucidation of this causal pathway could have important implications for policymakers interested in both physical and mental health improvement.

## Methods

### Data Availability

The data used for the observational analyses conducted in this study are available to qualified researchers upon application to the UK Biobank (https://www.ukbiobank.ac.uk/researchers/). The data used for the MR analyses conducted in this study are publicly available via the cited references. The scripts used to conduct the analyses are available at https://github.com/MRCIEU/education_mentalhealth_CVD/

### Observational Analyses

#### Sample

Our observational analyses used data from the UK Biobank, a national health resource that recruited participants aged 39 to 72 years from 22 centers across the UK between 2006 and 2010. Details of recruitment are publicly available.^17^ We excluded 814 individuals based on consent withdrawal, reported aneuploidy, or mismatch between reported and chromosomal sex. We then applied the Medical Research Council Integrative Epidemiology Unit quality control procedure to restrict the sample to individuals of European ancestry.^18^ The final sample size with complete phenotype measures was 333 525, with a mean age of 56.9 years (SD=8) and 54% women.

#### Measures

##### Educational Attainment

Participants in the UK Biobank reported the levels of qualifications attained and the highest level of qualification was converted into number of years in education using the International Standard Classification of Education coding of educational attainment (Table S1). Mean educational attainment was 13.9 years (SD=5.1). For regression analyses, educational attainment was categorized according to qualification level (minimal/none=0; further education=1; higher education=2) and thus odds ratios (ORs) should be interpreted as per increase in qualification level (approximately 3–5 years of additional attainment).

##### Mental Health Problems

Individuals were classified as having had a mental health problem if they reported “Yes” to either of the routine survey questions: “Have you ever seen a general practitioner (GP) for nerves, anxiety, tension, or depression?” or “Have you ever seen a psychiatrist for nerves, anxiety, tension, or depression?”

##### Depression

Individuals were classified as having major depressive disorder if their hospital episode statistics and/or general practice linked health record showed a diagnosis of a depressive episode or recurrent episodes according to the *International Classification of Diseases, Tenth Revision* (*ICD‐10*) codes F32 and F33.

##### Anxiety

Individuals were classified as having anxiety disorder if their health record showed such a diagnosis according to *International Classification of Diseases* (*ICD*) code F41.

The prevalence of these mediators in our sample was 34% for mental health problems, 2.9% for depression, and 1.4% for anxiety.

##### Cardiovascular Disease

Individuals were classified as having CVD if their health records showed at least 1 diagnosis of angina or myocardial infarction before recruitment classified by *ICD* codes I20 and I21, respectively. The prevalence of CVD in the sample at the time of recruitment was 6%.

Additional covariates included age, sex, smoking status, hypertension, obesity, and socioeconomic position. The methods for their classification and the respective prevalence figures are reported in Data S1 and Table S2.

### MR Analyses

#### Genetic Instruments

##### Educational Attainment

A total of 1271 independent genome‐wide significant loci (*P*<5×10^−8^) were identified in a sample of 1 131 881 individuals in a genome‐wide association study (GWAS) meta‐analysis of educational attainment by Lee and colleagues.^19^ Educational attainment was measured as years in education based on highest reported qualification level. The GWAS meta‐analyzed 71 cohorts of European ancestry measuring educational attainment. The median effect size of each allele of these lead variants was 1.7 weeks of schooling.

##### Mental Health Problems

Fourteen independent genome‐wide significant loci were identified in a recent GWAS of a phenotype described as “broad depression” in UK Biobank data (113 769 cases and 208 811).^20^ Here, we report this phenotype as “Having had a mental health problem” instead. Individuals were classified as having had a mental health problem if they reported “Yes” to either of the routine survey questions: “Have you ever seen a general practitioner (GP) for nerves, anxiety, tension, or depression?” or “Have you ever seen a psychiatrist for nerves, anxiety, tension, or depression?” Patients with diagnosed bipolar disorder or schizophrenia or who were taking antipsychotic medications were excluded. A number of participants were also excluded by interrelatedness filtering.

##### Depression

A total of 44 genome‐wide significant loci were identified in a GWAS meta‐analysis of major depressive disorder (135 458 cases and 344 901 controls) by Wray and colleagues.^21^ A total of 29 samples containing individuals of European ancestry were included in the analysis. Major depressive disorder was defined by meeting *Diagnostic and Statistical Manual of Mental Disorders (Fourth Edition)* (*DSM‐IV*), or *International Classification of Diseases, Ninth Revision/ICD*‐*10* criteria either by clinician review of medical records or by expert interview.

##### Anxiety

A recent GWAS of anxiety disorder (7016 cases and 14 745 controls) identified 1 independent genome‐wide significant locus.^22^ Anxiety cases were recruited from 7 different cohorts of European ancestry participating in the Anxiety NeuroGenetics Study with cases confirmed according to *DSM‐IV* criteria. To improve instrument power, we relaxed the significance threshold to *P*<5×10^−5^ and identified 108 independent significant loci following linkage disequilibrium clumping (*r* ^2^<0.001, distance >10 000 kb).

##### Cardiovascular Disease

We used summary data from a recent GWAS meta‐analysis from the CARDIoGRAMplusC4D (Coronary Artery Disease Genome‐Wide Replication and Meta‐Analysis [CARDIoGRAM] Plus the Coronary Artery Disease [C4D] Genetics) consortium.^23^ This included 48 cohorts with a total of 60 801 cases and 123 504 controls. The cohort studies typically defined a case as having a diagnosis of myocardial infarction or angina with several also requiring confirmation from angiographic evidence.

### Statistical Analysis

**Figure 1:**
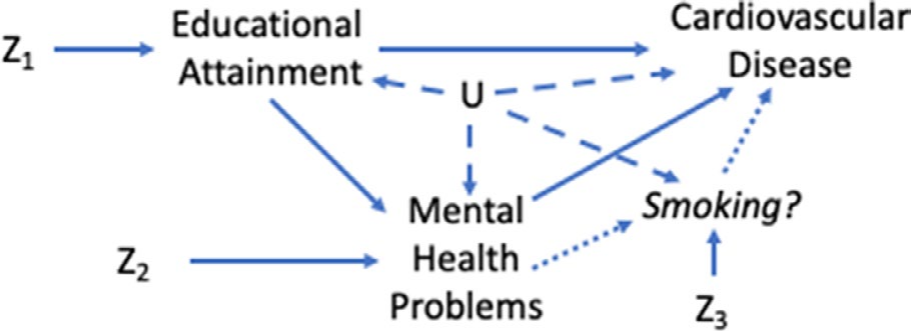
A diagram illustrating the proposed causal relationships between education, mental health, and cardiovascular disease. Z_1_, Z_2_ and Z_3_ represent genetic proxies and U represents confounding factors.

All analyses were performed using R (version 3.6.2).^24^ A directed acyclic graph for the proposed causal relationships investigated is shown in Figure 1.

#### Observational Analysis

First, we used logistic regression to investigate the association of educational attainment with CVD and with each of our mediators having adjusted for the effects of age and sex. Second, we analyzed the effect of each of our mediators on CVD and, in order to effectively isolate the mediator effects, adjusted for age, sex, educational attainment, smoking status, socioeconomic position, hypertension, and obesity. When specifically analyzing the effects of depression on CVD, we additionally adjusted for the effects of anxiety and vice versa.

A proportion‐mediated statistic was calculated using the product of coefficients method as detailed in previous work.^25^ The effect sizes were calculated using absolute risk differences as estimated from the ORs from our regression analysis and the known prevalence figures in UK Biobank. Standard errors were calculated using the delta method. *P* values were calculated using *Z* tests.

#### MR Univariable Analysis

We conducted 2‐sample MR analysis using the TwoSampleMR package 0.4.18.^26^ We first ran our analysis using educational attainment as an exposure and each of our proposed mediators and CVD as outcomes. We then repeated the analysis using each mediator as an exposure and CVD as an outcome.

For these analyses, we used an inverse‐variance weighted (IVW) approach as the main analysis,^27^ with additional statistical sensitivity analyses: weighted mode,^28^ weighted median,^29^ and MR‐Egger.^30^ Each sensitivity analysis makes different assumptions about pleiotropy, therefore a consistent estimate among all 4 methods provides the strongest evidence. Effect estimates produced were interpreted for directionality and, thus, whether they provided evidence against a causal null hypothesis.

When using the anxiety disorder instrument, we performed MR robust adjusted profile score in addition to IVW to improve estimation while using a relaxed *P* value threshold for the instrument.^31^ MR robust adjusted profile score offers a greater robustness to pleiotropy when using many weak instruments.

#### Two‐Step MR Analysis

We next performed 2‐step MR using the TwoSampleMR package 0.4.18. We used the results of our univariable MR analysis for the exposure‐mediator step. For the mediator‐outcome step we then used the residual multivariable MR method to calculate the effect of each mediator on CVD having adjusted for the effects of educational attainment.^32^ The estimates for each step were multiplied together to produce an indirect effect estimate and the proportion‐mediated statistic was then calculated as for the observational analysis (detailed above).

### Sensitivity Analyses

The methods for our sensitivity analyses are presented in Data S1. These analyses included MR‐Egger, Cochran Q Tests of Heterogeneity, mean of F statistics, MR Steiger, and bidirectional observational models. They also included an observational analysis of mental health and CVD conducted without adjustment for other covariates except for age and sex. Sensitivity plots were also included for genetic instruments displaying susceptibility to bias from pleiotropy as well as MR analyses displaying significant heterogeneity.

### Exploratory Analysis

To illustrate an approach for investigating multiple mediators between mental health and CVD, we performed an exploratory analysis of a likely candidate based on existing evidence. This analysis investigates the role of tobacco smoking as a mediator between depression and CVD. The methods are in Data S1.

#### Ethical Considerations

UK Biobank has received ethics approval from the UK National Health Service’s National Research Ethics Service (reference 11/NW/0382).

## Results

### Association of Education With CVD

**Figure 2:**
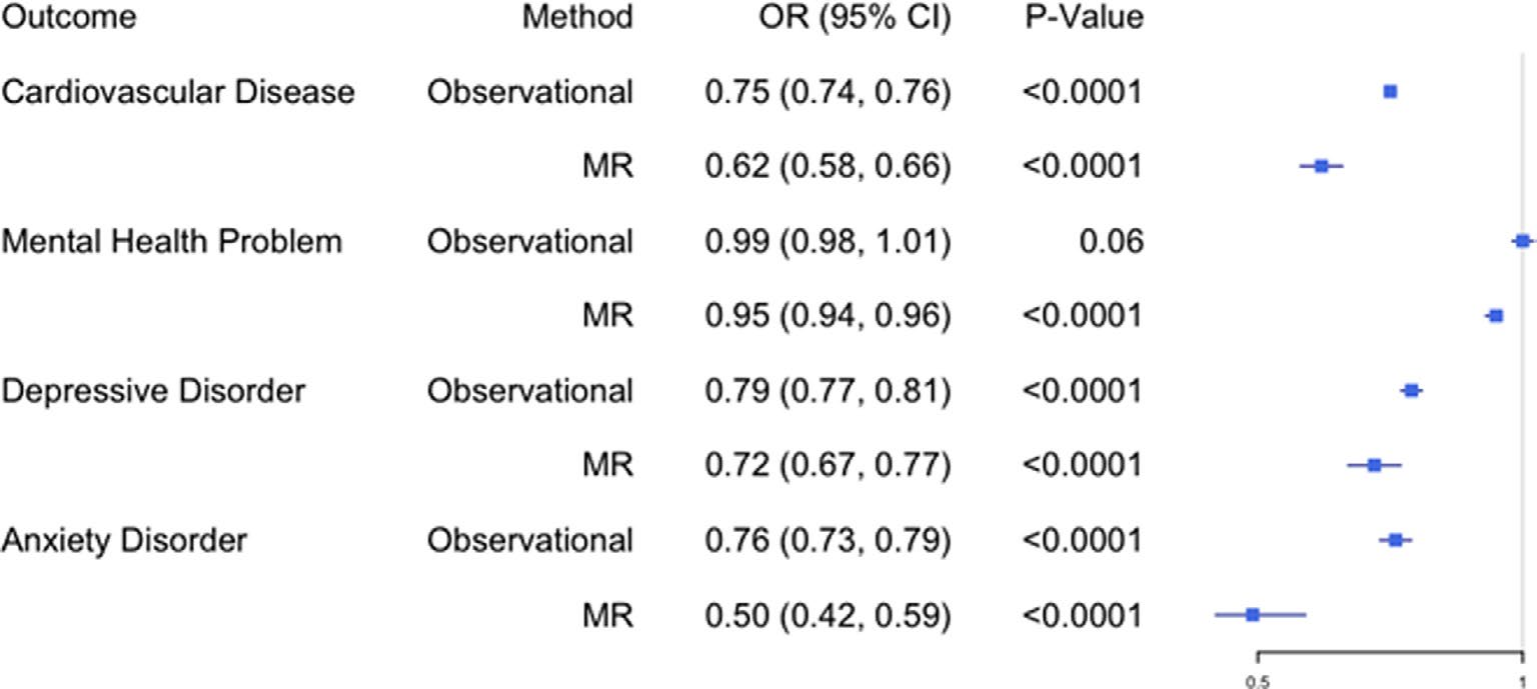
The association of educational attainment with mental health and cardiovascular disease presented as odds ratios (ORs) per qualification level increase in attainment. MR indicates Mendelian randomization.

We found strong evidence to suggest that educational attainment has an inverse association with CVD (Figure 2). In our observational analysis, increased educational attainment was associated with a reduction in CVD (OR, 0.75; 95% CI, 0.74–0.76 per qualification level increase in attainment). Our MR analysis provided evidence for a causal, protective effect of education on CVD (IVW OR, 0.62; 95% CI, 0.58–0.66).

### Association of Education With Mental Health

We also found that education has an inverse association with anxiety and depression and that this is consistent among both adjusted observational and MR analyses (Figure 2). Increased educational attainment was associated with reductions in depression and anxiety (OR, 0.79 [95% CI, 0.77–0.81] and 0.76 [95% CI, 0.73–0.79], respectively, per qualification level increase in attainment). Our MR analysis provided evidence for education having a causal, protective effect against anxiety and depression (IVW OR, 0.50 [95% CI, 0.42–0.59] and 0.72 [95% CI, 0.67–0.77], respectively).

Educational attainment generally showed only weak evidence of an association with mental health problems in our observational analysis (OR, 0.99; 95% CI, 0.98–1.01 [*P*=0.06]) but, in our MR analysis, there was some evidence of a causal effect (IVW OR, 0.95; 95% CI, 0.94–0.96 [*P*<0.0001]). This association was consistent among the MR sensitivity analyses.

### Association of Mental Health With CVD

**Figure 3:**
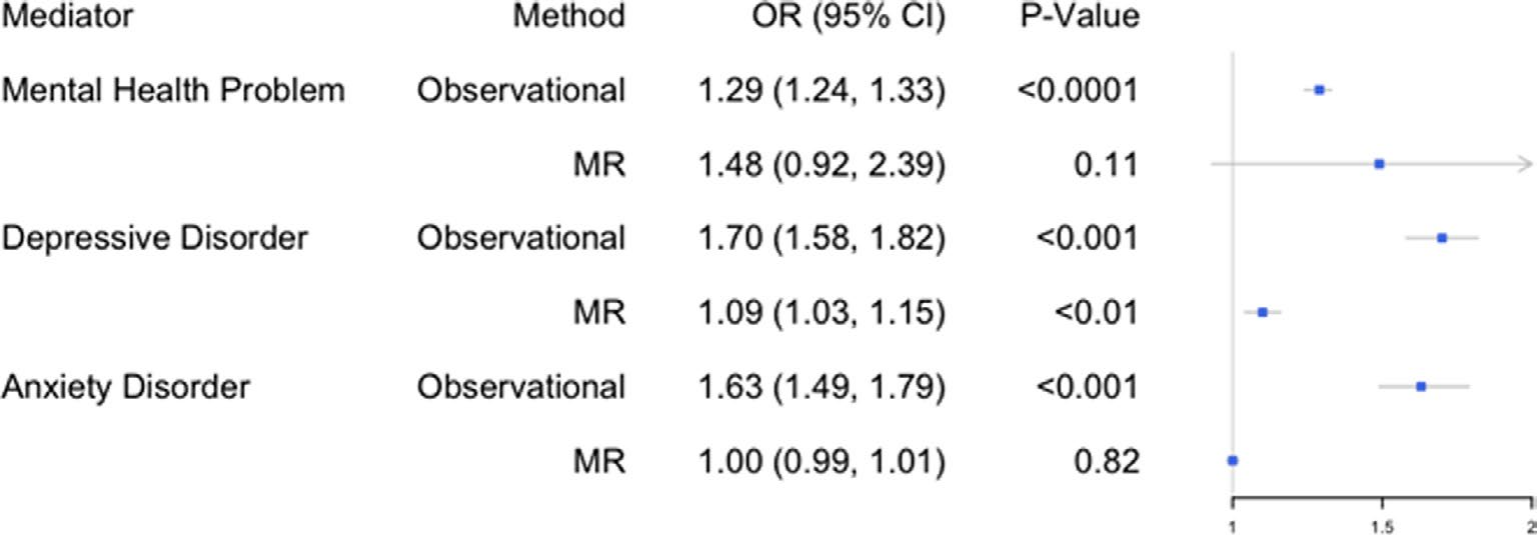
The association of mental health with cardiovascular disease following adjustment for the effects of educational attainment. OR indicates odds ratio; and MR, Mendelian randomization.

In our observational analysis, mental health problems in general were associated with increased CVD risk (OR, 1.29; 95% CI, 1.24–1.33 [*P*<0.001]), but our MR analysis did not provide clear support for a causal interpretation (2‐step MR: OR, 1.48; 95% CI, 0.92–2.39 [*P*=0.11]) (Figure 3).

However, we found strong observational evidence that depression was associated with increased cardiovascular risk (OR, 1.70; 95% CI, 1.58–1.82 [*P*<0.001]), and our MR analyses provided evidence for a causal effect (2‐step MR: OR, 1.09; 95% CI, 1.03–1.15 [*P*=0.0021]). Anxiety disorder was strongly associated with CVD in our observational analysis (OR, 1.63; 95% CI, 1.49–1.79 [*P*<0.001]) but MR analysis did not support a causal interpretation (2‐step MR: OR, 1.00; 95% CI, 0.99–1.01 [*P*=0.82]).

### Proportion of the Association Between Education and CVD Mediated By Mental Health

**Figure 4:**
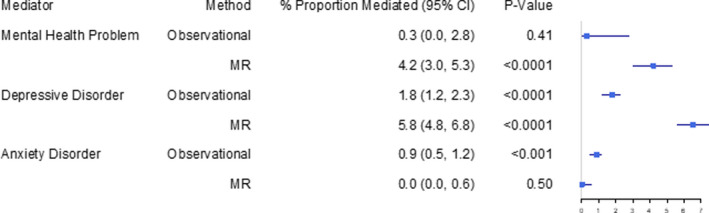
The estimated proportions of the inverse association between education and cardiovascular disease accounted for by mental health problems, depression, and anxiety. MR indicates Mendelian randomization.

**Figure 5:**
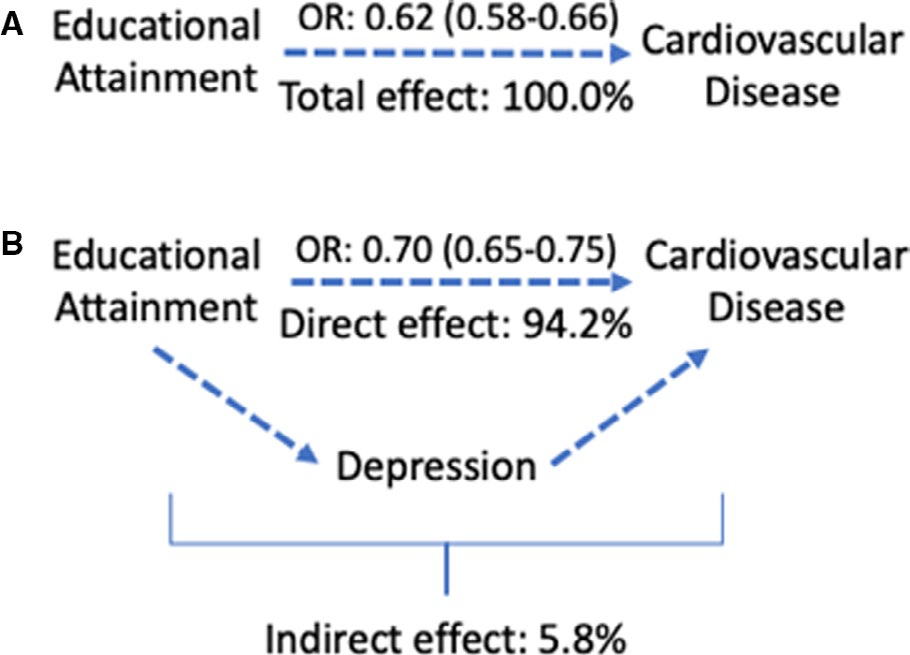
A diagram illustrating the breakdown of (**A)** the total effect of educational attainment on cardiovascular disease into (**B**) a direct effect and an indirect effect via depression. OR indicates odds ratio (with 95% CI). Estimates are from Mendelian randomization (MR) analysis.

Our mediation results show that mental health problems in general (observational: 0.3% [95% CI, 0.0%–2.8%]; 2‐step MR: 4.2% [95% CI, 3.0%–5.3%]) and specifically depression (observational: 1.8% [95% CI, 1.2%–2.3%]; 2‐step MR: 5.8% [95% CI, 4.8%–6.8%]) account for a small proportion of the negative association between education and CVD (Figures 4 and 5). The proportion mediated by anxiety disorder was small in the observational analysis, and we found little evidence of mediation in our MR analysis (observational: 0.9% [95% CI, 0.0%–0.6%]; 2‐step MR: 0.0% [95% CI, 0.0%–0.6%]).

### Sensitivity Analysis

The results of our sensitivity analyses are presented in Tables S3 through S13 and included testing for pleiotropy and reverse causation, heterogeneity, bidirectionality in our observational data, and personality traits as additional potential mediators. The results did raise the possibility of reverse causation between CVD and the proposed mediators in our observational analyses. There was also evidence of horizontal pleiotropy for the educational attainment and lifetime smoking instruments, which both displayed disparities between IVW and MR‐Egger results. The MR‐Egger intercept also showed weak evidence of directional horizontal pleiotropy for the lifetime smoking instrument (OR, 1.01; 95% CI, 1.00–1.02 [*P*=0.02]) although no other MR‐Egger intercepts did. Finally, several of the analyses, particularly those utilizing educational attainment as an exposure, displayed significant effect heterogeneity.

### Exploratory Analysis of Tobacco Smoking as a Mediator Between Depression and CVD

**Figure 6:**
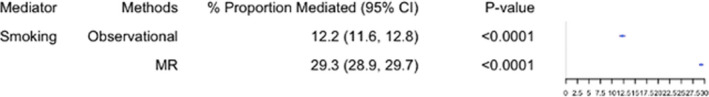
The estimated proportion of the association between depression and cardiovascular disease accounted for by lifetime smoking. MR indicates Mendelian randomization.

**Figure 7:**
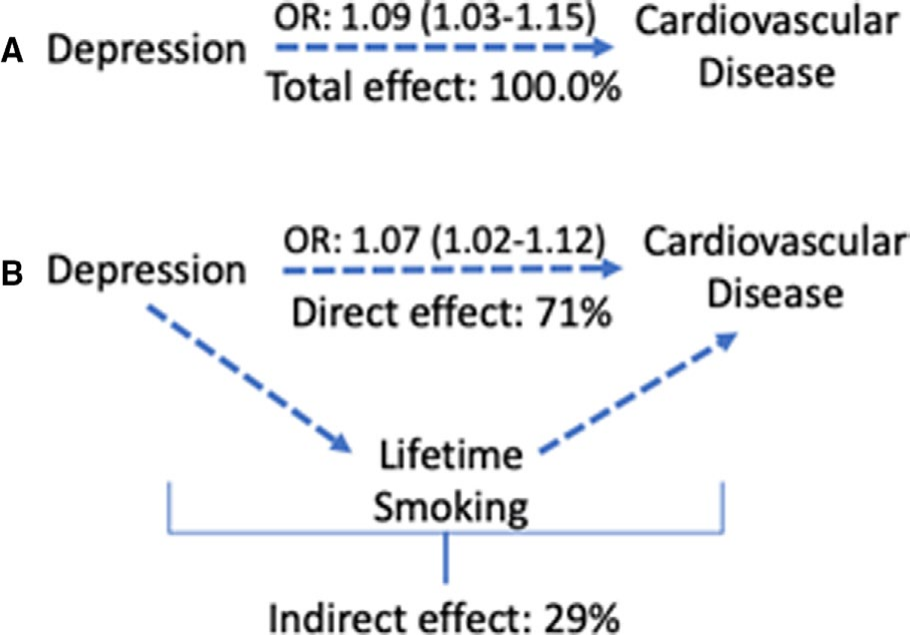
A diagram illustrating the breakdown of (**A**) the total effect of depression on cardiovascular disease into (**B**) a direct effect and an indirect effect via smoking. OR indicates odds ratio (with 95% CI). Estimates are from Mendelian randomization analysis.

In exploratory analysis, we investigated smoking as a possible mediator on the pathway from depression to CVD. We found that depression is associated with increased smoking activity and that lifetime smoking was associated with increased CVD risk (Figures S1 and S2). In Figures 6 and 7, we show that smoking accounts for a large proportion of the increased CVD risk associated with depression (observational: 12.2% [95% CI, 11.6%–12.8%]; 2‐step MR: 29% [95% CI, 28.9%–29.7%]).

## Discussion

In this study, we used both observational and MR techniques to investigate mental health as a mediator between education and CVD. Our results demonstrate that depression accounts for a proportion of the protective effect education has on CVD.

We demonstrated, in observational analyses, that educational attainment has a clear, inverse association with anxiety disorder, major depressive disorder, and CVD. Our MR analyses provided evidence for these relationships being causal. In addition, our MR analyses provided evidence for a causal, protective effect of educational attainment on mental health problems more broadly although observational analysis showed no strong association. Mental health problems in general and, specifically, anxiety and depression were all associated with increased cardiovascular risk in our observational analyses. Our MR analyses provided evidence only for causality between depression and CVD.

Following these initial results, we further explored the proposed causal relationships between education, mental health, and CVD, which likely consist of additional mediators (Figure 1). Our observational analyses showed that depression was positively associated with lifetime smoking, which, in turn, was associated with increased cardiovascular risk even after adjustment for depression. Our MR analyses provided evidence for these associations being causal. We found that lifetime smoking accounts for as much as 30% of the association between depression and CVD.

### Our Results in Context

Our analysis suggests that depression serves as a mediator between education and CVD, and thus accounts for an unexplained portion of the protective effect of education. Specifically, it suggests that educational disparities may produce additional CVD burden partly by increasing rates of depression. Previous MR analyses found no clear association between education and depression^33^ and this was likely the result of weak instrument strength. Using stronger instruments in the current study, we did see novel evidence for a causal effect. In contrast, depression and coronary artery disease have previously been found to be associated using MR work,^34^, ^35^ and our mediation analysis constitutes a novel finding that can help identify possible new targets for interventions.

Our results support a protective role for education against mental health problems and, specifically, major depressive disorder and anxiety disorder. The foremost explanation for this is educational attainment being a proxy for overall socioeconomic position, which has a robust negative correlation with depression and anxiety.^36^ For example, educational attainment is associated with both higher income and better occupation later in life.^37^, ^38^ Such factors protect against experiences such as debt, financial struggles, and poor working conditions, which feature among several relevant risk factors for mental health disorders.^39^ Related interventions would accordingly include policies to promote educational attainment and reduce socioeconomic inequalities.

At an individual level, further years spent in education or training are also likely to play a protective role against mental health problems by directly modifying a range of psychosocial factors and providing resources that can be drawn on as stressors are encountered.^40^ Such resources might include mental health advice, support networks, social participation, close friends, and good work‐life balance.^41^, ^42^ Additional years in education may also help to offset exposure to stressors until one has greater maturity and resources to cope with them. By considering these observations, successful individual‐level interventions in the education system might be developed.

In an exploratory analysis, we found that smoking accounts for a large proportion of the association between depression and CVD. The associations between smoking and CVD are well‐known.^43^, ^44^ The association between smoking and depression has been commonly attributed to a “self‐medication” hypothesis where smoking is used to cope with depressive symptoms. Other factors that are proposed to partially explain the remainder of the association between depression and CVD include chronic inflammation and changes in lipid metabolism.^45^

### Implications

Our study has several important public health implications. First, it strengthens the case for investment in education interventions by demonstrating that educational attainment can reduce mental health burden, which is itself a significant source of morbidity and mortality.^46^ Second, by illustrating the links between education, mental health, and CVD, we have reinforced the need to recognize the potential interplay between social determinants and physical and mental health problems. Indeed, our study shows how a physical disease burden could be partly alleviated by targeting a social determinant of mental health and provides further support for the use of existing mental health interventions to reduce risk for CVD.^47^, ^48^ Finally, we have also illustrated how to further explore potential mediators between depression and CVD in order to elucidate additional intervention targets. Here, we have highlighted tobacco smoking as such a target.

### Strengths and Limitations

The strengths of our study include the use of MR analysis to complement a conventional observational approach, which allowed us to better overcome bias arising from confounding, measurement error, and reverse causation. The use of a “triangulation approach” also allowed for greater confidence in causal inference when there was consistency between the different approaches.^49^ We were also able to limit bias arising from self‐reporting and misclassification by using strict case definitions for both our observational and MR data sources. Although more relaxed case definitions may have improved the generalizability of results, potential bias arising from their use can be particularly problematic when using binary exposures in MR.^50^ An additional strength of our study was the use of 2‐step MR for a mediation analysis. This allowed us to specifically quantify the proportion of our exposure’s effect attributable to our proposed mediators. Finally, we extended our analysis to illustrate how our methodology could explore multiple mediators in the proposed pathway.

In our study, the use of MR demonstrated a discrepancy when observing the effects of mental health on CVD. When adjusting for confounding in our mediation analysis, we likely overadjusted in order to confidently isolate the effects of the mediator. To measure this, we conducted a sensitivity analysis without adjustment, although this suggested that the effect sizes we have found may actually be underestimates (Table S14). Instead, the discrepancy may have primarily been caused by reverse causation, with CVD leading to mental health problems and exaggerating observed associations. In a further sensitivity analysis, where CVD was the exposure and depression and then anxiety the outcomes, we did indeed find such bidirectionality (Table S15).

A common limitation of MR studies is weak instrument strength,^51^ especially when strict case definitions are used. Our instruments for both educational attainment and depression were adequate in this regard, with large mean of F statistics and sample sizes. However, our instrument for anxiety had a relatively smaller mean of F statistic and required a relaxed genome‐wide significance threshold; the null result should therefore be interpreted with caution and the analysis repeated when better MR instruments for anxiety are available. However, we utilized the MR robust adjusted profile score method to compensate for the relaxed threshold and we also conducted sensitivity analysis with the worry subscale of the Eysenck Personality Questionnaire (Revised Short Form). This analysis had adequate instrument strength and a larger sample size but also suggested no clear evidence (Table S8).

Despite adequate instrument strength, our “mental health problems” instrument produced wide CIs, suggesting that these analyses may have been underpowered. Unlike our other instruments, this one was constructed from a GWAS performed on a single sample, as the unusual phenotype measured is only available in the UK Biobank. The phenotype is also not clearly defined and is more heterogeneous than phenotypes using specific definitions for depression and anxiety. We believe this reiterates the need for future work to focus on generating instruments with larger sample sizes and specific phenotype definitions.

When conducting 2‐sample MR, overlap between the samples can be problematic. The samples used for our exposure and mediators did feature partial overlap attributable to the use of UK Biobank data in both sets. This introduces a greater risk of a type I error in our 2‐sample univariable analyses of education and mental health caused by possible overfitting. However, we also saw an inverse association with education in our sensitivity analyses that utilized personality traits related to mental health (Table S8). These featured substantively less overlap between samples. In addition, this limitation did not apply to our 2‐step analysis, which pools the data for the exposure and mediators before analysis is conducted.

Another common limitation of MR studies is bias from directional pleiotropy.^52^ We conducted numerous sensitivity analyses, including multiple MR methods and measurement of MR‐Egger intercepts to assess for these. These suggested that the instruments for educational attainment and lifetime smoking could be biased by horizontal pleiotropy and that several related analyses also displayed effect heterogeneity. However, the direction of estimates was still largely consistent among a range of MR methods with sensitivity plots also demonstrating no specific variants driving these particular observations (Figures S3 through S12).

Despite this, caution should still be employed. Furthermore, the generalizability of our results is also limited given that our genetic instruments were solely constructed from cohorts of White European ancestry and that UK Biobank participants specifically skew to higher educational levels. This is in addition to another known issue with MR studies, namely that the genetic compliers producing the effect estimates may only constitute a subpopulation of the sample that may not be representative. Our instrument for educational attainment was based on a meta‐analysis of several different populations, which would have helped to mitigate against these issues; however, our other genetic instruments were not as comprehensive. These limitations ultimately reiterate the need to interpret our MR results as evidence against a causal null hypothesis rather than as precise causal effect estimates. In the future, we hope that alternative genetic instruments based on a diverse range of subpopulations will be available to redress the issues highlighted.

Finally, even with our approach, education may still be acting as a proxy for socioeconomic position. A proposed strategy for investigating this would be to conduct an additional analysis using a measure such as household income, which would allow for comparison as well as adjustment as part of a multivariable MR approach. A recently published GWAS meta‐analysis exploring household income^53^ offers the possibility of this in future studies. Natural experiment techniques could also be used for this purpose as well as to support our results, having previously suggested that the raising of the school‐leaving age reduced CVD.^54^

## Conclusions

We have demonstrated that educational attainment is inversely associated with mental health problems, anxiety, depression, and CVD. A reduction in depression accounts for part of the inverse association between education and CVD. Finally, we have shown that smoking accounts for a large proportion of the association between depression and CVD. Our findings offer significant clinical and public health implications by further demonstrating the interplay between social determinants, mental and physical disease, and associated targets for intervention.

## Sources of Funding

R.E.W., H.M.S., A.R.C., D.G., and M.R.M. are all members of the Medical Research Council (MRC) Integrative Epidemiology Unit at the University of Bristol funded by the MRC: http://www.mrc.ac.uk [MC_UU_00011/7]. D.G. is supported by the Wellcome Trust 4i Programme (203928/Z/16/Z) and British Heart Foundation Research Centre of Excellence (RE/18/4/34215) at Imperial College London, and by a National Institute for Health Research Clinical Lectureship (CL‐2020‐16‐001) at St. George's, University of London. This study was supported by the National Institute Health Research (NIHR) Biomedical Research Centre at the University Hospitals Bristol National Health Service (NHS) Foundation Trust and the University of Bristol. The views expressed in this publication are those of the authors and not necessarily those of the NHS, NIHR, or Department of Health and Social Care.

## Disclosures

D.G. is employed part‐time by Novo Nordisk. The remaining authors have no disclosures to report.

## Supporting information

Data S1: Supplementary Methods
Table S1–S15
Figure S1–S12
References 55, 56, 57, 58, 59, 60
