# Supplementary material for: Mental Health as a Mediator of the Association Between Educational Inequality and Cardiovascular Disease: A Mendelian Randomization Study
Source: J Am Heart Assoc. 2021 Sep 2;10(17):e019340. doi: 10.1161/JAHA.120.019340 (PMC8649303; doi:10.1161/JAHA.120.019340)

Supplemental Material

Data S1.

Supplemental Methods

Methods for co-variates used in observational analysis

For age and sex, individuals were classified according to reported age and sex at time of recruitment. Individual smoking status was classified according to whether an individual reported never smoking, being a previous smoker or being a current smoker. Individuals were classified as having hypertension if their health records showed a diagnosis as classified by ICD code I10 and obesity if their records showed a diagnosis as classified by ICD code E66. Socioeconomic position was estimated using Townsend Deprivation Index (TDI) at birth.

Methods for sensitivity analyses

Additional MR tests: The regression dilution I^2 statistic was used to assess the suitability of the genetic instrument for use in the MR Egger method[55]. If the I^2 statistic was less than 0.9, then SIMEX corrections were conducted. This statistic also serves to measure potential bias arising from measurement error with values close to 1 indicating minimal attenuation. We also assessed the strength of the genetic instruments using the Mean F-statistic, with a value above 10 indicating adequate instrument strength[56]. In addition, we conducted a Cochran's Q-test to assess for heterogeneity[57] and then utilised the MR Egger intercept in order to assess for directional pleiotropy. We also applied Steiger filtering directionality tests to assess for reverse causation by identifying genetic variants that explain more variance in the outcome than the exposure[58].

Bidirectionality: As a sensitivity analysis, we investigated the directionality of these effects by measuring the effect of cardiovascular disease on our proposed mediators having adjusted for age, sex and SES.

Additional mediators: We also investigated whether personality traits such as depressed effect and worry were mediating the relationship between educational attainment and cardiovascular disease, rather than mental health problems. These traits were depressed affect and worry with instruments created based on summary data from a recent GWAS meta-analysis of neuroticism[59]. This included 12 cohorts with a total of 449,484 individuals and identified 136 independent genome-wide significant loci for neuroticism, depressed affect and worry.

Smoking as a mediator between mental health problems and cardiovascular disease: Finally, we investigated whether smoking behaviours further mediated the pathway from mental health problems to cardiovascular disease using multivariable regression and MR approaches. For the observational analyses, we used UK Biobank data and classified participants on a never vs ever smoker basis. For the MR analyses, we constructed an instrument for smoking initiation using summary data from the GSCAN GWAS[60] which defined smoking initiation as ever having smoked more than 100 cigarettes, smoking every day for at least a month or smoking regularly. The sample size was 1,232,091 and it identified 378 independent genome-wide significant genetic loci which explain 4% of the variance in smoking initiation. We used the same summary data reporting cardiovascular disease for our outcome as before.

We utilised the product of co-efficients method as described in our main methods to calculate the proportion mediated statistic. Effects were estimated using logistic regression for our traditional observational data and two-step MR for our genetic instruments. In the first step when calculating the indirect effects, we adjusted for age, sex, educational attainment, obesity, socio-economic position and hypertension in our logistic regression. In the second step, we adjusted for age, sex and depression in the logistic regression and depression only in the two-step MR. Standard errors were calculated using the delta method.

Table S1. International Standard for Classification of Education (ISCED) codes applied to levels of qualification reported in UK biobank data.

**Highest Level of Qualification**	**ISCED Code**	**Years of full-time Education**
College/University Degree	5	20
National Vocational Qualification/equivalent	5	19
Other professional qualification	4	15
A-levels/AS/Equivalent	3	13
GCSE/CSE/O-Level/Equivalent	2	10
None of the above	1	7
Prefer not to say	N/A	Excluded

Table S2. Reported demographics in UK Biobank for co-variates utilised in observational analysis.

**Co-Variate**		**Demographics**
Age in years (mean (SD))		56.9 (8)
Sex (%)	Male	46
	Female	54
Smoking Status (%)	Never	55.2
	Former	35.5
	Current	9.3
Hypertension (%)		18.7
Obesity (%)		2.6
Townsend Deprivation Index (mean (SD))		-1.58 (2.93)

Table S3. Full results of MR univariable analysis.

Exposure	Outcome	Method	SNPs	OR (95% CI)	P-value
Educational Attainment	Cardiovascular Disease	Inverse Variance Weighted (IVW)	1206	0.62 (0.58-0.66)	3.5×10^{-50}
		MR Egger Simex (Unweighted)	1206	0.73 (0.58-0.91)	0.004
		Weighted Median	1206	0.64 (0.59-0.70)	1.1×10^{-24}
		Weighted Mode	1206	0.71 (0.47-1.06)	0.10
	Major depressive Disorder (MDD)	IVW	1068	0.72 (0.67-0.77)	8.0×10^{-22}
		MR Egger Simex (Unweighted)	1068	0.62 (0.51-0.75)	1.8×10^{-6}
		Weighted Median	1068	0.75 (0.69-0.81)	4.9×10^{-12}
		Weighted Mode	1068	0.98 (0.61-1.57)	0.94
	Anxiety Disorder	IVW	1151	0.50 (0.42-0.59)	8.8×10^{-16}
		MR Egger Simex (Weighted)	1151	0.49 (0.21-1.14)	0.10
		Weighted Median	1151	0.50 (0.39-0.66)	3.5×10^{-7}
		Weighted Mode	1151	0.60 (0.21-1.70)	0.33
	Mental Health Problems	IVW	568	0.95 (0.94-0.96)	7×10^{-12}
		MR Egger Simex (Unweighted)	568	0.97 (0.93-1.02)	0.20
		Weighted Median	568	0.95 (0.93-0.96)	7.2×10^{-11}
		Weighted Mode	568	0.91 (0.86-0.97)	0.002
MDD	Cardiovascular Disease	IVW	39	1.09 (0.98-1.22)	0.13
		Weighted Median	39	1.10 (0.95-1.28)	0.20
		Weighted Mode	39	1.20 (0.90-1.61)	0.23
Anxiety		IVW	94	1.01 (0.99-1.02)	0.27
		MR Egger Simex (Unweighted)	94	1.01 (0.98-1.04)	0.42
		Weighted Median	94	1.01 (0.99-1.03)	0.29
		Weighted Mode	94	1.01 (0.98-1.04)	0.59
		MR RAPS	94	1.01 (1.00-1.03)	0.13
Mental Health Problems		IVW	13	2.68 (1.32-5.44)	0.01
		Weighted Median	13	2.79 (1.06-7.33)	0.04
		Weighted Mode	13	2.41 (0.42-13.80)	0.34
MDD	Lifetime Smoking	IVW	35	1.07 (1.03-1.12)	1.5×10^{-3}
		Weighted Median	35	1.07 (1.04-1.10)	8.5×10^{-6}
		Weighted Mode	35	1.08 (1.02-1.14)	9.6×10^{-3}
Lifetime Smoking	Cardiovascular Disease	IVW	120	1.87 (1.49-2.36)	9.3×10^{-8}
		MR Egger Simex (Unweighted)	120	0.68 (0.31-1.53)	0.35
		Weighted Median	120	2.07 (1.53-2.79)	2.1×10^{-6}
		Weighted Mode	120	2.35 (1.00-5.52)	0.05

Table S4. Regression dilution I^2_{GX} to test the suitability of instruments for MR Egger and the mF statistic as a test of instrument strength for MR univariable analysis.

Exposure	Outcome	mF	I^2 Unweighted G_X	I^2 Weighted G_X
Educational Attainment	Cardiovascular disease	46.66	0.732	0.568
	Major Depressive Disorder (MDD)	46.64	0.736	0.556
	Anxiety	47.10	0.689	0.766
	Mental Health Presentation	48.37	0.770	0.574
MDD	Cardiovascular disease	36.63	0	0
Anxiety		18.51	0.524	0
Mental Health Problems		36.80	0	0
MDD	Lifetime Smoking	37.95	0	0
Lifetime Smoking	Cardiovascular disease	44.05	0.644	0.454

Table S5. The number of SNPs from the genetic instruments in the MR univariable analysis that explain more of the variance in the exposure than the outcome according to Steiger directionality tests.

Exposure	Outcome	True (%)	FALSE
Educational Attainment	Cardiovascular disease	1206 (95.3)	60
	Mental health problems	595 (100)	0
	Major Depressive Disorder (MDD)	1228 (96.6)	51
	Anxiety	702 (58.2)	505
Mental health problems	Cardiovascular disease	12 (92.3)	1
MDD		40 (100)	0
Anxiety		95 (100)	0
MDD	Lifetime Smoking	36 (100)	0
Lifetime Smoking	Cardiovascular Disease	116 (92.1)	10

Table S6. Cochran's Q Tests of Heterogeneity for MR univariable analysis.

Exposure	Outcome	IVW Q (Degrees of freedom)	IVQ Q P-value
Educational Attainment	Cardiovascular Disease	1499.43 (1205)	<0.001
	Mental Health Problems	1193.86 (567)	<0.001
	Major Depressive Disorder (MDD)	1901.98 (1067)	<0.001
	Anxiety	1111.07 (1150)	0.79
Mental Health Problems	Cardiovascular disease	11.72 (12)	0.47
MDD		47.81 (38)	0.13
Anxiety		95.85 (93)	0.40
MDD	Lifetime Smoking	261.97 (34)	<0.001
Lifetime Smoking	Cardiovascular disease	183.08 (119)	<0.001

Table S7. MR Egger Intercept test of directional horizontal pleiotropy for MR univariable analysis.

Exposure	Outcome	MR Egger Intercept	Standard Error	P-value
Educational Attainment	Cardiovascular Disease	-0.00144	0.00132	0.28
	Mental Health Problems	-0.000193	0.000286	0.50
	Major Depressive Disorder (MDD)	0.000999	0.00138	0.47
	Anxiety	-0.00197	0.00378	0.60
Mental Health Problems	Cardiovascular Disease	0.0193	0.0183	0.31
MDD		0.00729	0.00125	0.56
Anxiety		-0.000687	0.00293	0.82
MDD	Lifetime Smoking	0.00367	0.00479	0.45
Lifetime Smoking	Cardiovascular Disease	0.0122	0.00496	0.02

Table S8. Results of MR univariable analysis utilising personality traits.

Exposure	Outcome	Method	SNPs	OR (95% CI)	P-value
Educational Attainment	Depressed Affect	Inverse Variance Weighted (IVW)	1140	0.75 (0.73-0.77)	1.2x10^{-133}
		MR Egger	1140	0.74 (0.69-0.80)	5.9x10^{13}
		Weighted Median	1140	0.77 (0.59-0.70)	1.2x10^{-104}
		Weighted Mode	1140	0.76 (0.67-0.87)	6.3x10^{-5}
	Worry	IVW	1140	0.92 (0.90-0.94)	4.3x10^{-11}
		MR Egger	1140	0.96 (0.88-1.04)	0.32
		Weighted Median	1140	0.92 (0.89-0.94)	8.9x10^{-12}
		Weighted Mode	1140	0.87 (0.78-0.98)	0.02
Depressed Affect	Cardiovascular Disease	IVW	54	1.49 (1.18-1.87)	0.0007
		MR Egger	54	1.36 (0.41-4.50)	0.61
		Weighted Median	54	1.38 (1.07-1.77)	0.01
		Weighted Mode	54	1.41 (0.79-2.52)	0.26
Worry		IVW	54	0.95 (0.75-1.21)	0.68
		MR Egger	54	0.65 (0.18-2.36)	0.51
		Weighted Median	54	1.08 (0.83-1.41)	0.55
		Weighted Mode	54	1.14 (0.64-2.01)	0.66

Table S9. Results of multivariable MR analysis utilising personality traits with odds ratios for each mediator adjusted for effects of exposure presented.

Exposure	Mediator	Outcome	OR (95% CI)	P-value
Educational Attainment	Worry	Cardiovascular Disease	1.04 (0.97-1.11)	0.54
	Depressed Affect		1.10 (0.99-1.21)	0.06

Table S10. Cochran's Q Tests of Heterogeneity for multivariate MR analysis.

Exposure	Mediator	Outcome	Q (Degrees of freedom)	Q P-value
Educational Attainment	Major Depressive Disorder (MDD)	Cardiovascular Disease	1534 (1269)	<0.001
	Anxiety		1677 (1271)	<0.001
	Mental Health Problems		1782 (1329)	<0.001
Educational Attainment	MDD	Lifetime Smoking	3129 (1084)	<0.001
MDD	Lifetime Smoking	Cardiovascular Disease	189 (141)	<0.01

Table S11. MR Egger Intercept test of directional horizontal pleiotropy for MR multivariable analysis.

Exposure	Mediator	Outcome	MR Egger Intercept	Standard Error	P-value
Educational Attainment	Mental Health Problems	Cardiovascular Disease	0.00104	0.000532	0.05
	Major Depressive Disorder (MDD)		0.000555	0.000357	0.12
	Anxiety		0.000405	0.000354	0.25
Educational Attainment	MDD	Lifetime Smoking	0.0000204	0.0000851	0.81
MDD	Lifetime Smoking	Cardiovascular Disease	0.00236	0.00102	0.02

Table S12. The MR Egger mF statistic as a test of instrument strength in MR multivariable analysis.

Exposures	Mediator	Outcome	mF	P-value
Educational Attainment	Mental Health Problems	Cardiovascular disease	60.81	<0.001
	Major Depressive Disorder (MDD)		117.9	<0.001
	Anxiety		113.4	<0.001
Educational Attainment	MDD	Lifetime Smoking	533.9	<0.001
MDD	Lifetime Smoking	Cardiovascular disease	19.8	<0.001

Table S13. Results of MR univariable analysis of personality traits.

Exposure	Outcome	Method	SNPs	OR (95% CI)	P-value
Educational Attainment	Depressed Affect	Inverse Variance Weighted (IVW)	1140	0.75 (0.73-0.77)	$1.2x10^{-133}$
		MR Egger	1140	0.74 (0.69-0.80)	$5.9x10^{13}$
		Weighted Median	1140	0.77 (0.59-0.70)	$1.2x10^{-104}$
		Weighted Mode	1140	0.76 (0.67-0.87)	$6.3x10^{-5}$
Educational Attainment	Worry	IVW	1140	0.92 (0.90-0.94)	$4.3x10^{-11}$
		MR Egger	1140	0.96 (0.88-1.04)	0.32
		Weighted Median	1140	0.92 (0.89-0.94)	$8.9x10^{-12}$
		Weighted Mode	1140	0.87 (0.78-0.98)	0.02
Depressed Affect	Cardiovascular Disease	IVW	54	1.49 (1.18-1.87)	0.0007

Table S14. Results of logistic regression of mental health on cardiovascular disease with adjustment for age, sex and educational attainment only.

Mediator	Outcome	OR (95% CI)	P-value
Mental health problems	Cardiovascular disease	1.54 (1.48-1.60)	8.63×10^{-129}
Major depressive disorder		2.77 (2.60-2.96)	2.60×10^{-212}
Anxiety disorder		2.39 (2.19-2.61)	2.27×10^{-84}

\

Table S15. Results of logistic regression assessing the effect of cardiovascular disease on mental health utilising observational data after adjustment for co-variates.

Exposure	Outcome	OR (95% CI)	P-value
Cardiovascular Disease	Mental Health Problems	1.23 (1.19-1.26)	$1.2x10^{-31}$
	Anxiety Disorder	1.55 (1.40-1.72)	$3.5x10^{-21}$
	Major Depressive Disorder	1.57 (1.46-1.68)	$9.1x10^{-38}$

Figure S1. Results of univariable observational and MR analyses investigating association of depression with lifetime smoking index (LSI).

Exposure	Outcome	Method	Mean Difference (95% CI)	P-Value
Depression	LSI	Observational	+0.23 (0.22, 0.24)	<0.0001
		MR	+0.04 (0.03, 0.05)	<0.01

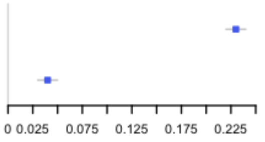

Figure S2. Results of multivariable observational and MR analyses investigating association of lifetime smoking with cardiovascular disease having adjusted for depression.

Mediator	Outcome	Method	OR (95% CI)	P-Value
LSI	CVD	Observational	1.32 (1.29, 1.34)	<0.0001
		MR	1.87 (1.49, 2.36)	<0.0001

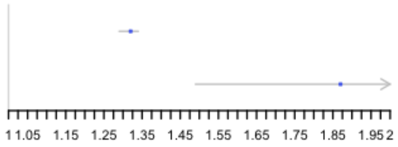

Figure S3. Plot of MR leave-one-out analysis of educational attainment and cardiovascular disease.

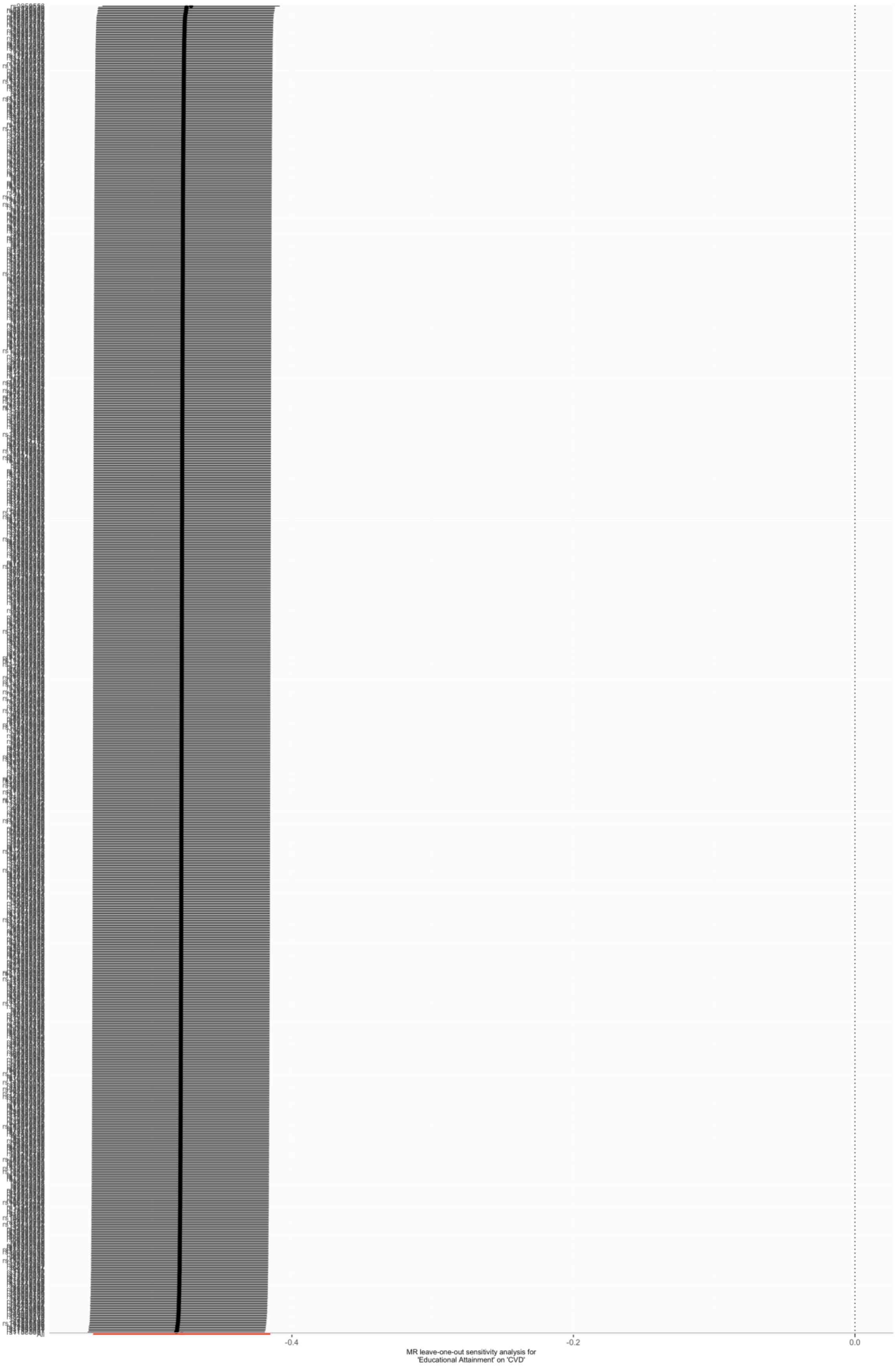

Figure S4. Scatter plot of MR analysis of educational attainment and cardiovascular disease.

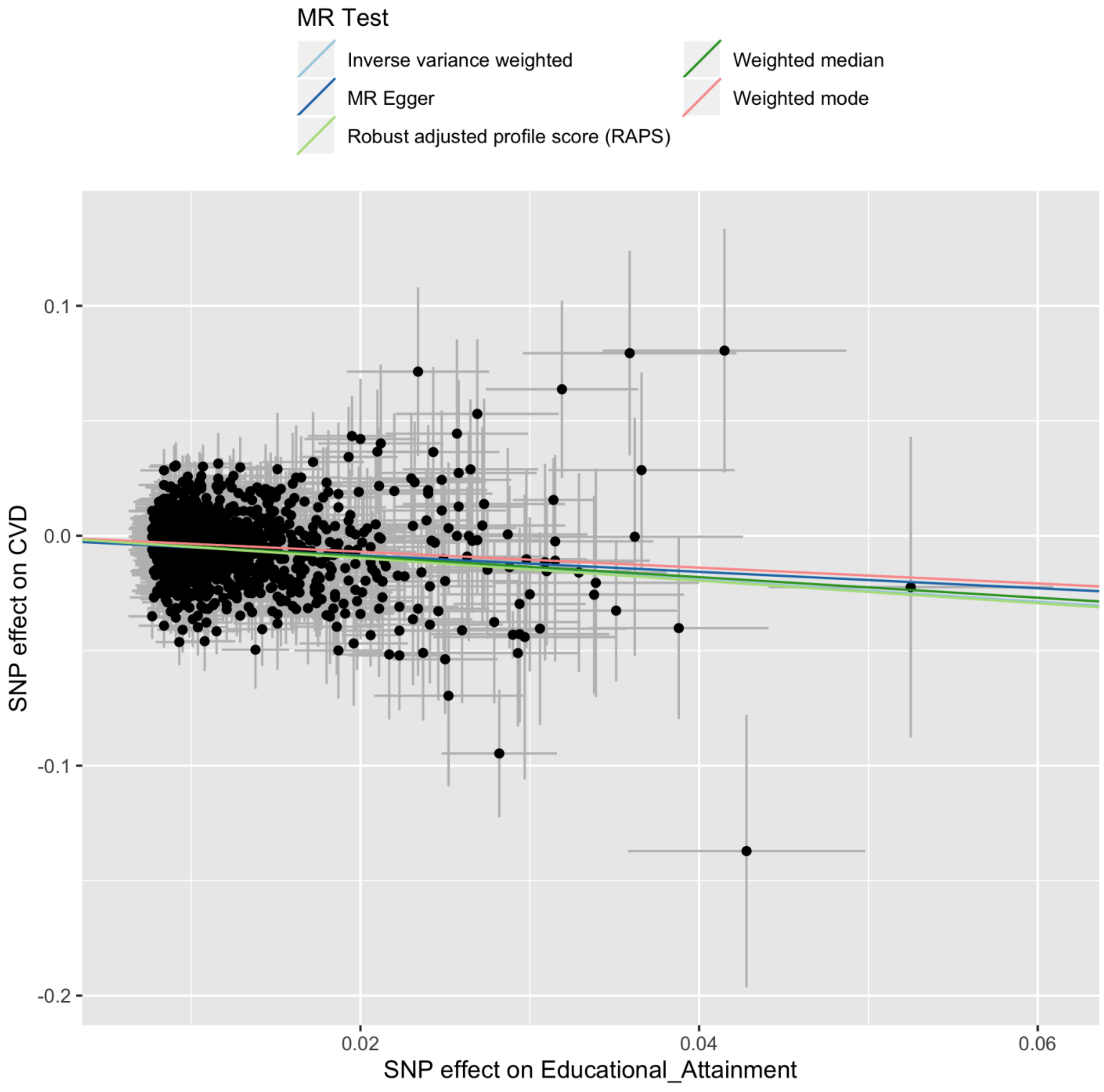

Figure S5. Plot of MR leave-one-out analysis of educational attainment and mental health problems.

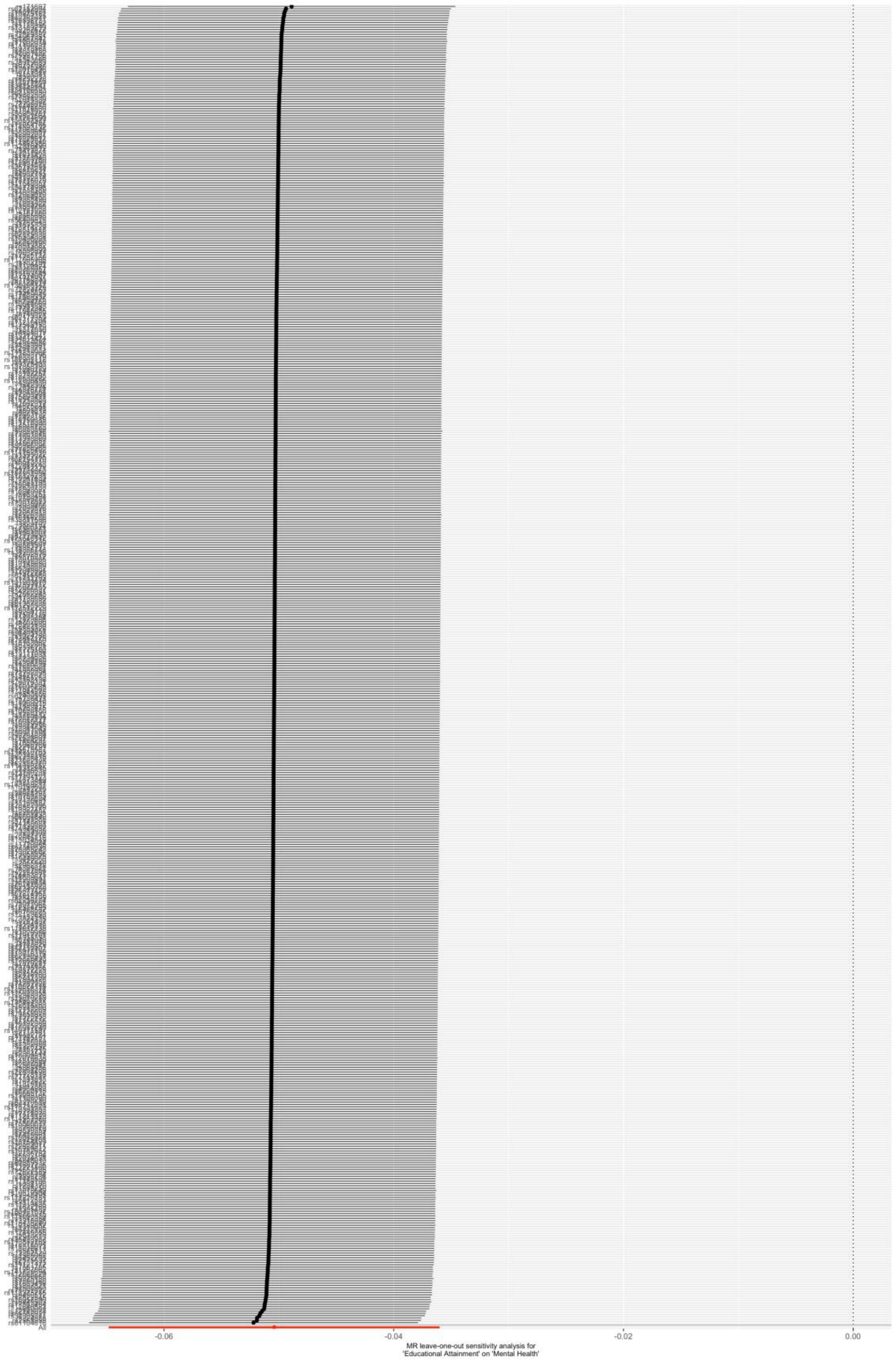

Figure S6. Scatter plot of MR analysis of educational attainment and mental health problems.

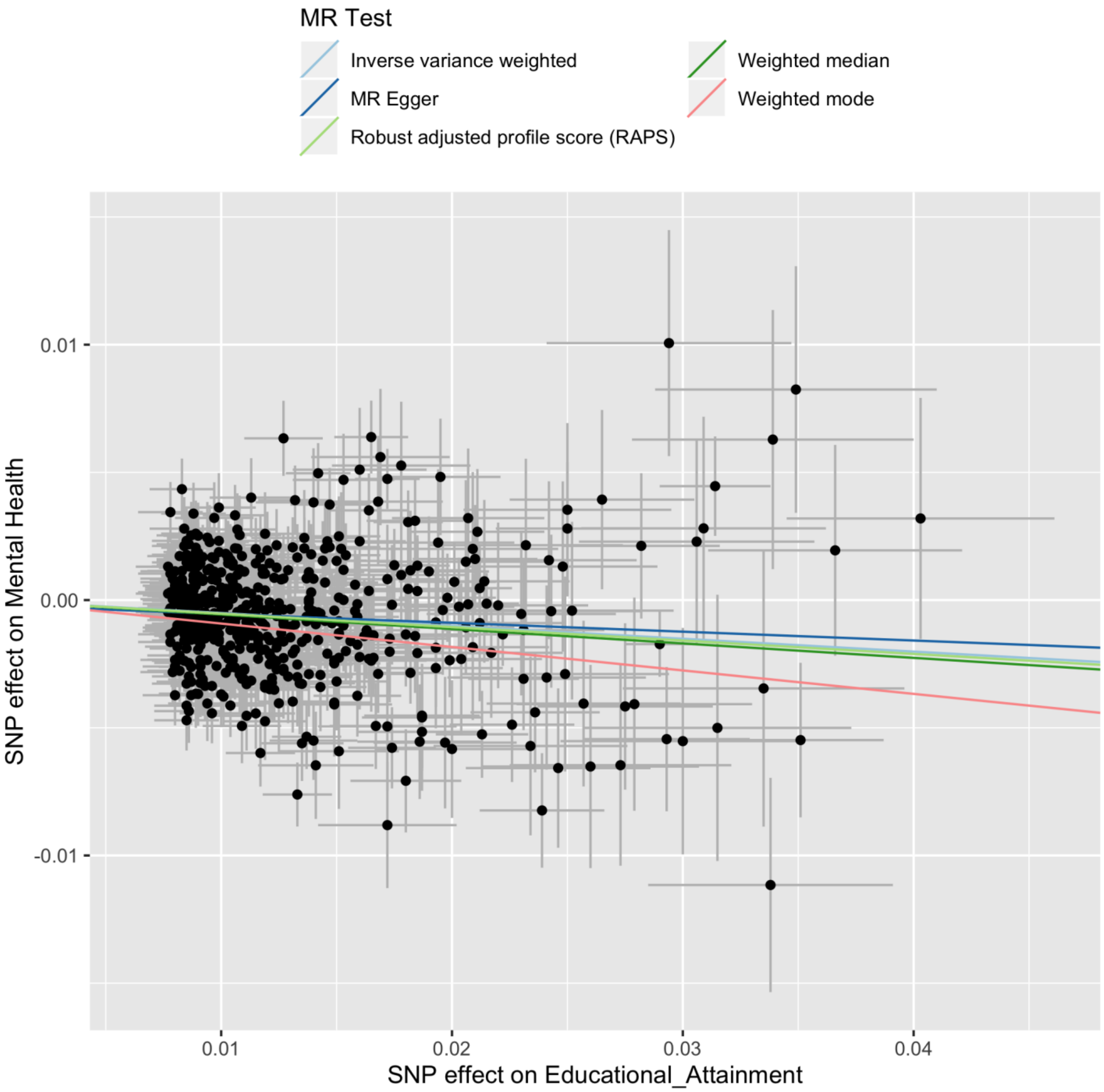

Figure S7. Plot of MR leave-one-out analysis of educational attainment and depression.

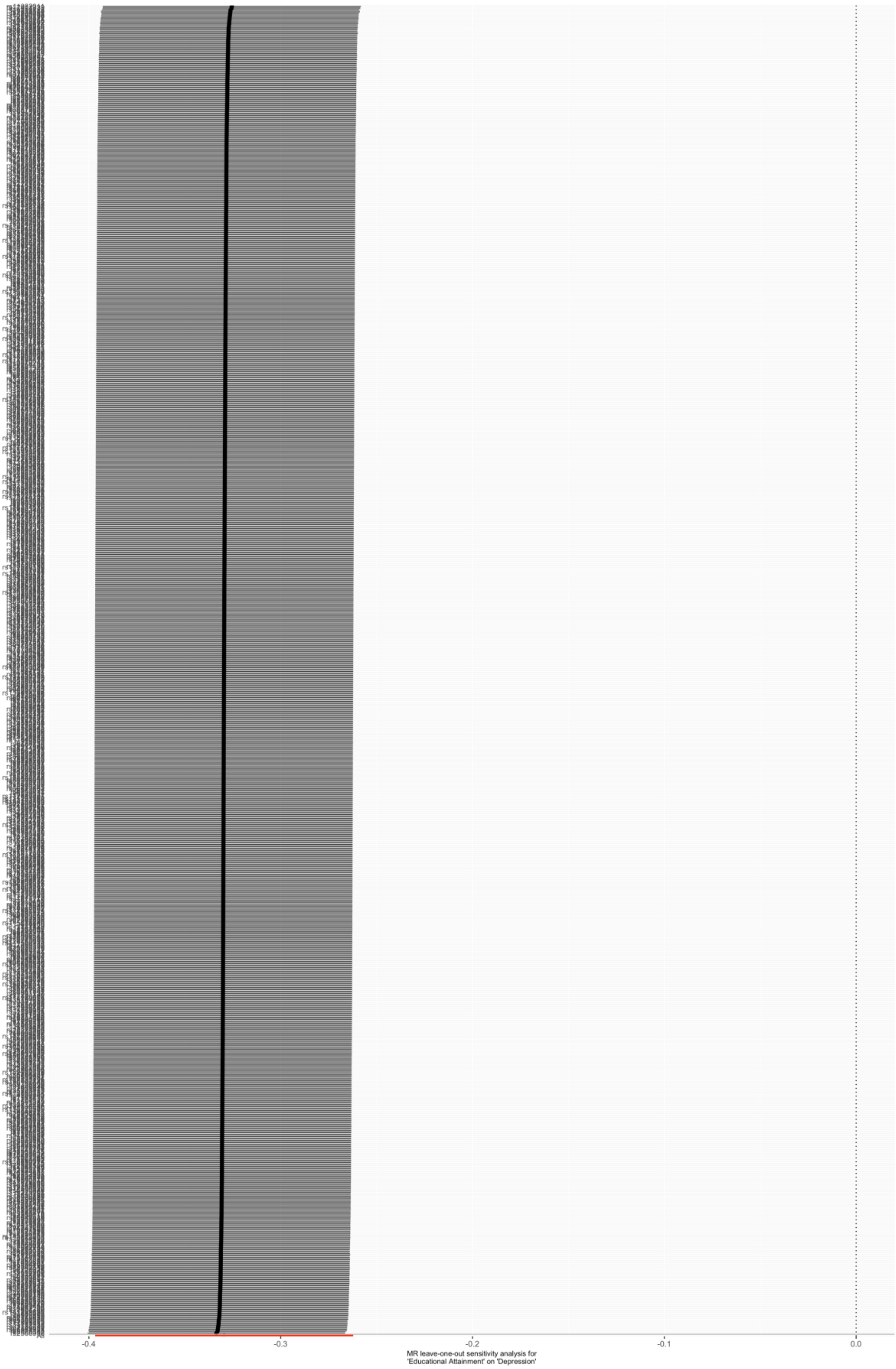

Figure S8. Scatter plot of MR analysis of educational attainment and depression.

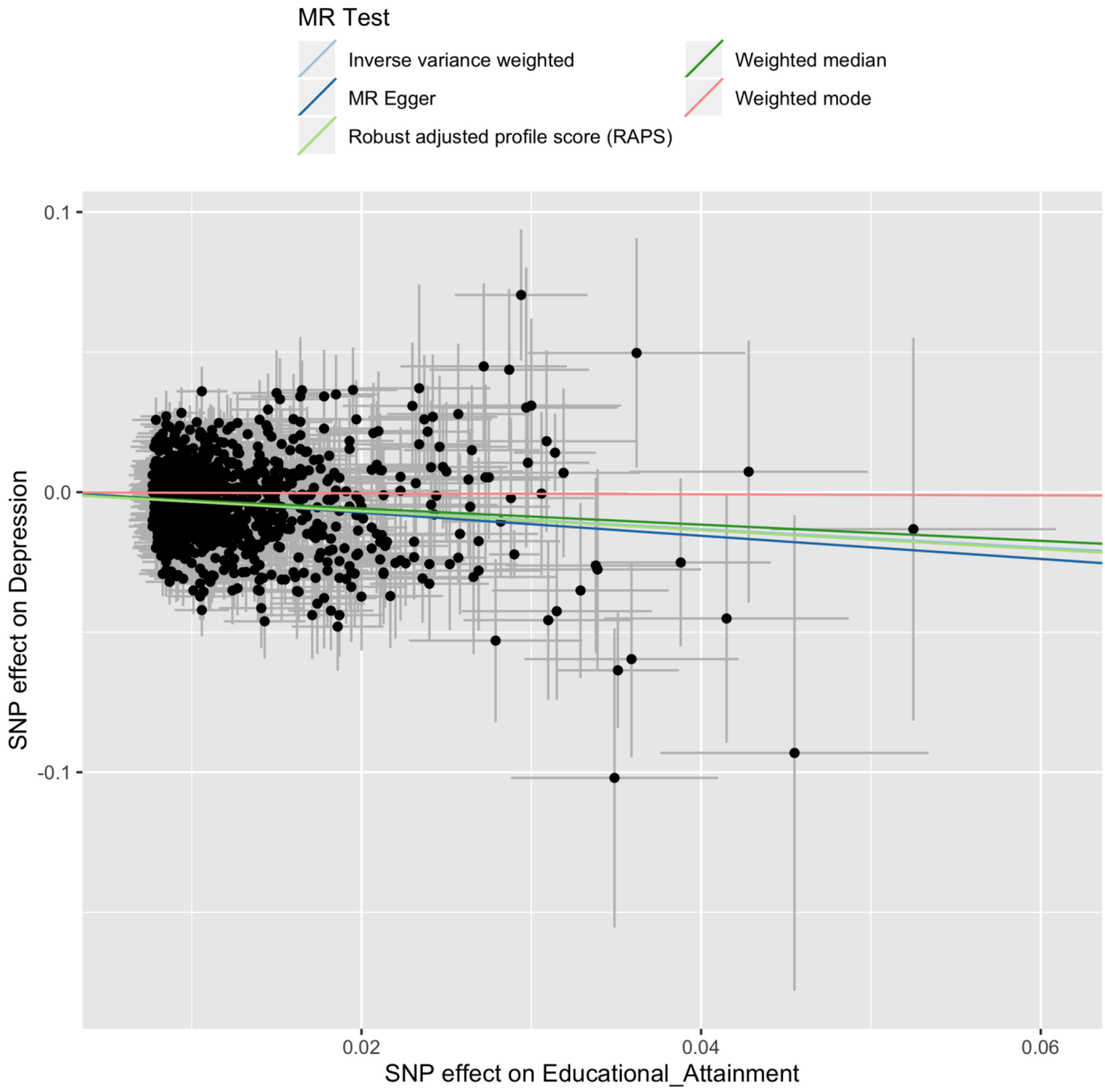

Figure S9. Plot of MR leave-one-out analysis of depression and lifetime smoking.

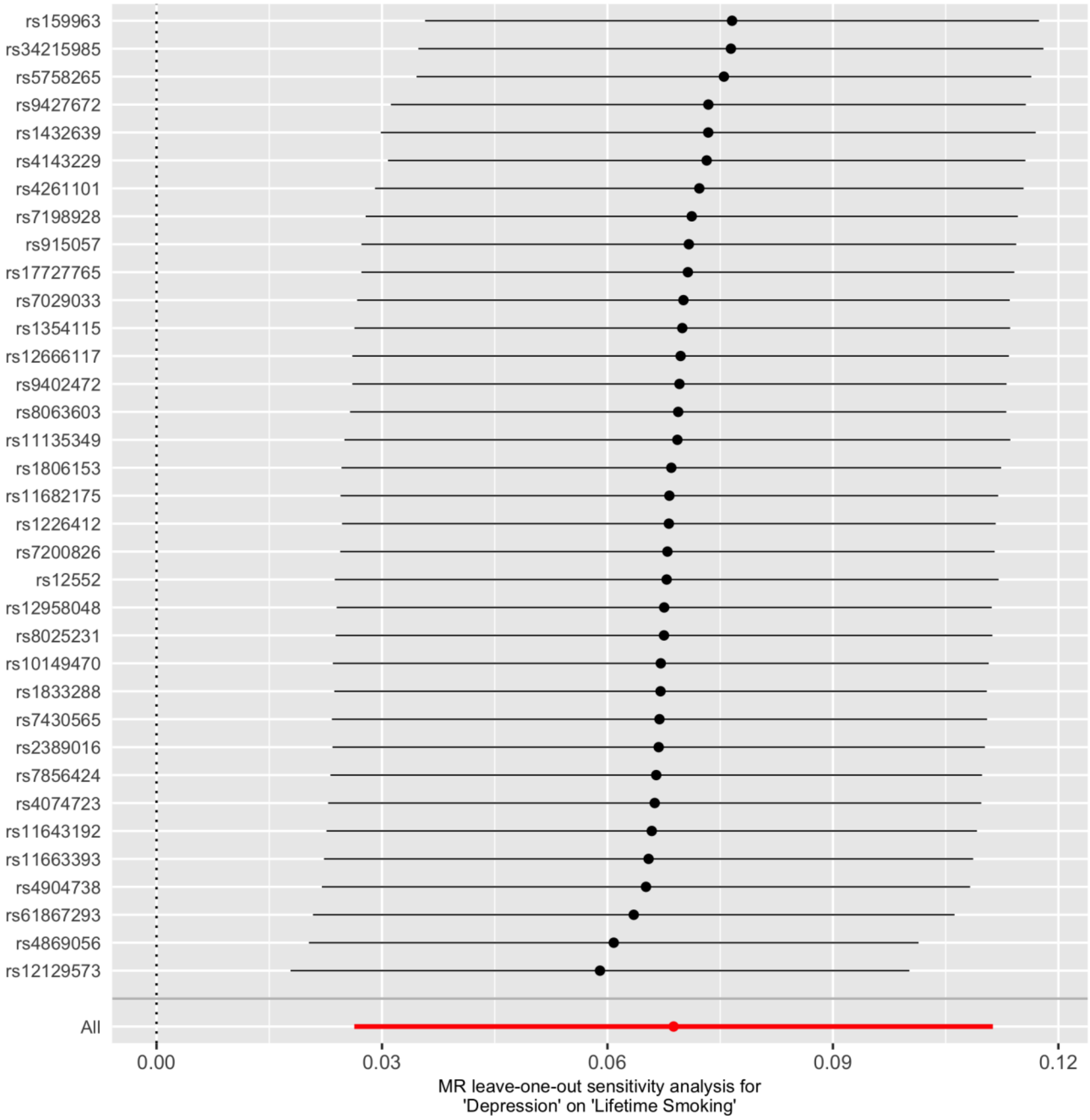

Figure S10. Scatter plot of MR analysis of depression and lifetime smoking.

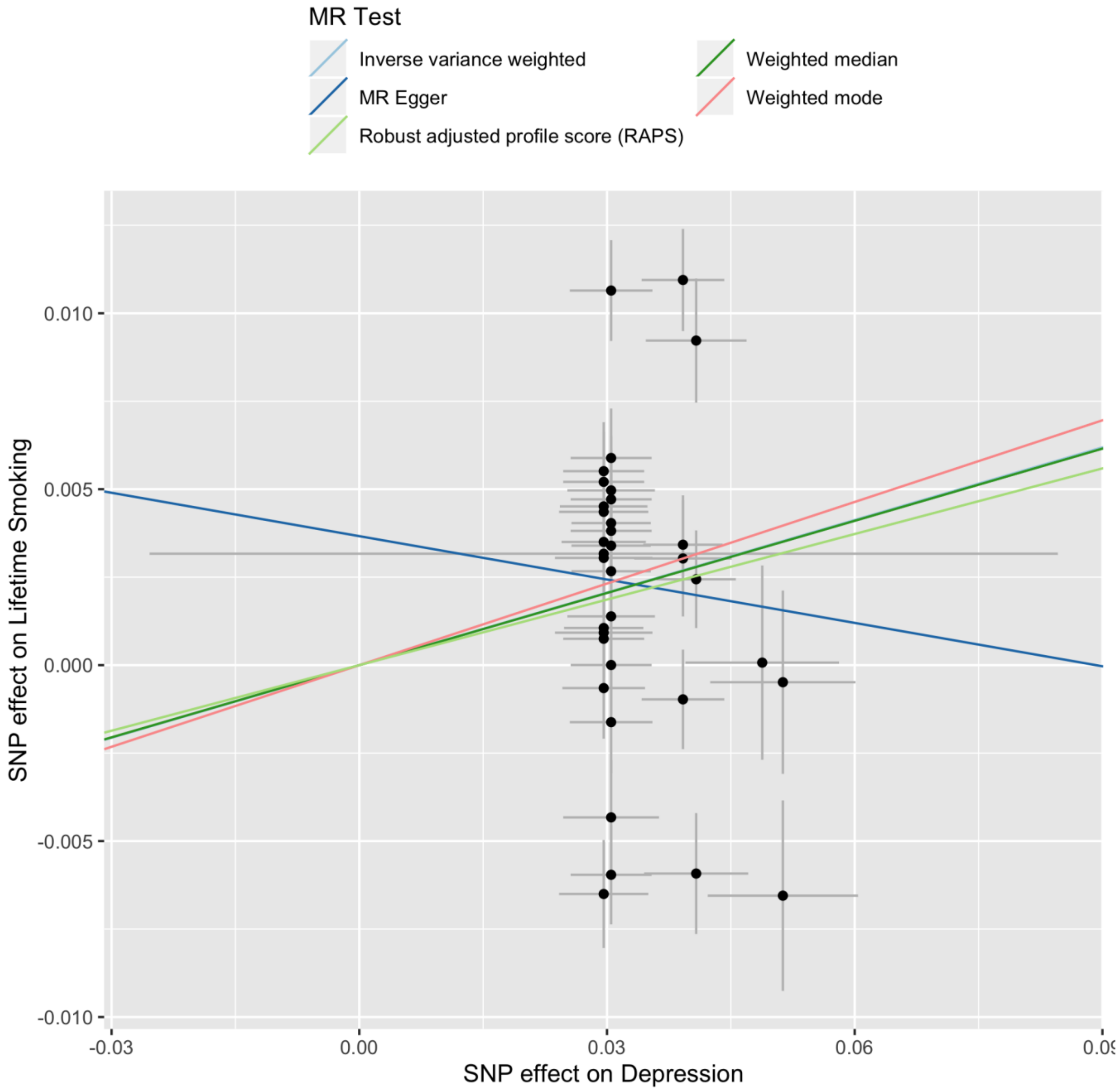

Figure S11. Plot of MR leave-one-out analysis of lifetime smoking and cardiovascular disease.

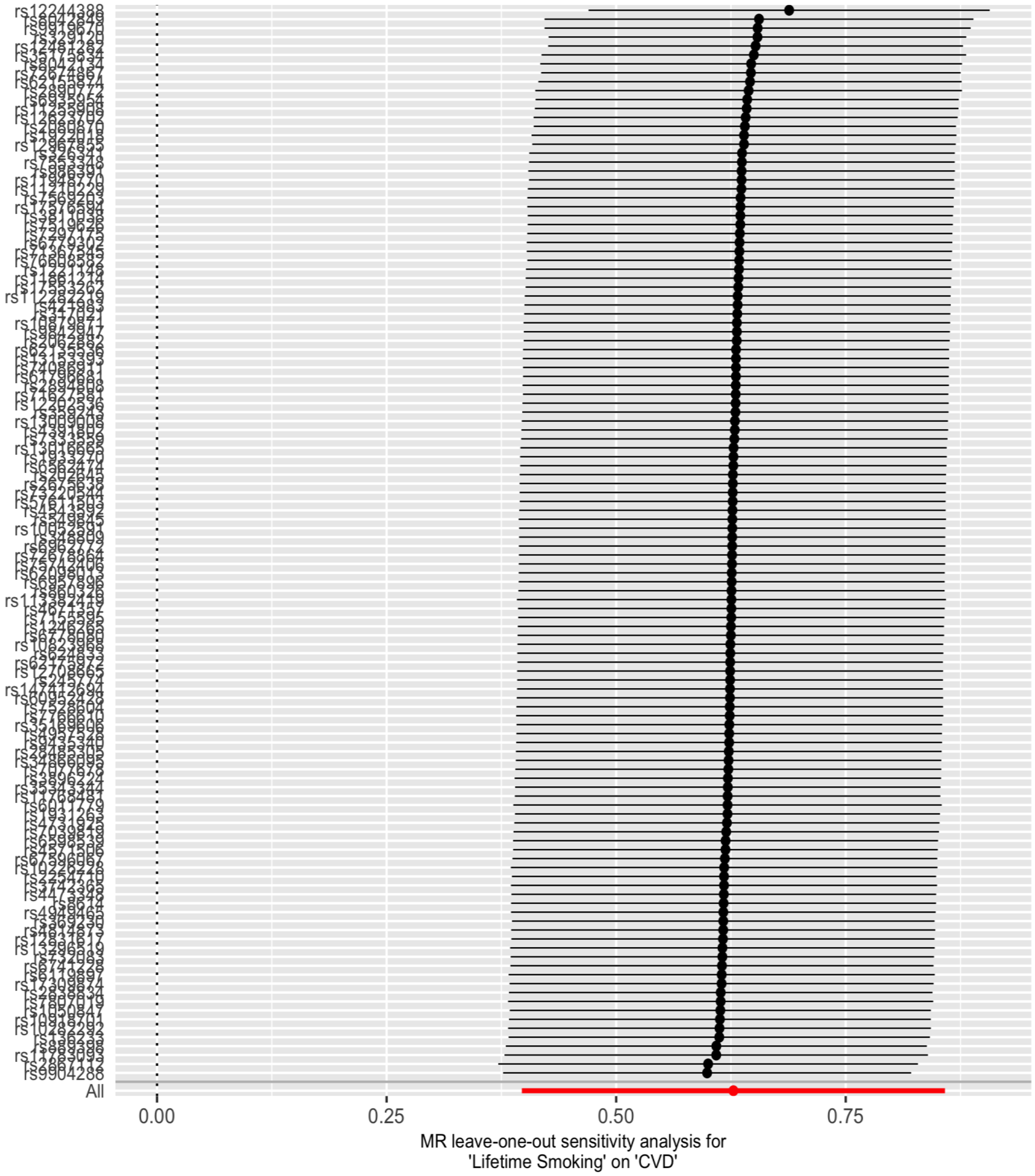

Figure S12. Scatter plot of MR analysis of lifetime smoking and cardiovascular disease.

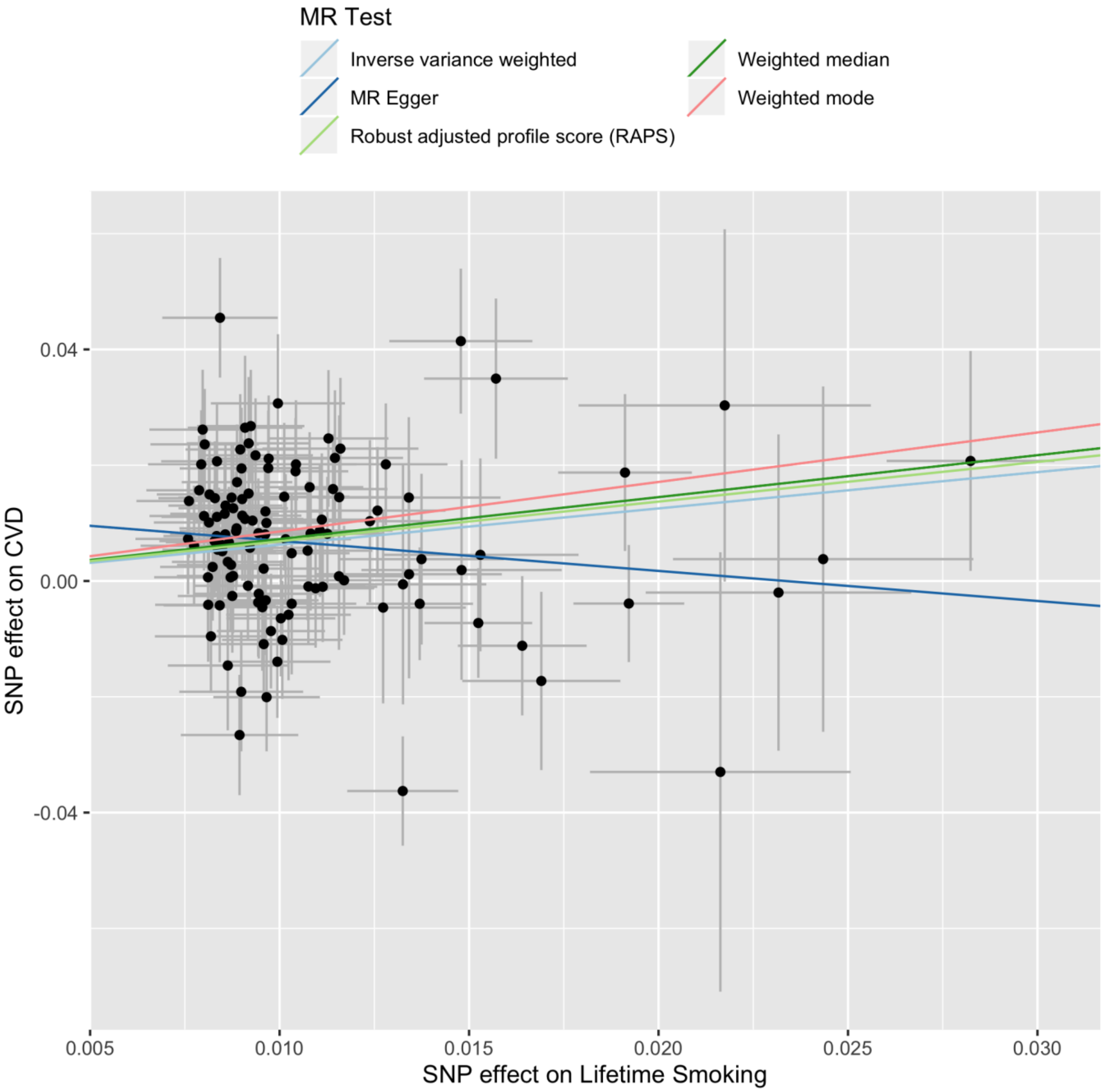

Supplement: Supplementary file 1 — Data S1: Supplementary Methods Table S1–S15 Figure S1–S12 References 55, 56, 57, 58, 59, 60 [file JAH3-10-e019340-s001.pdf]
